# A Study of the Detection of SARS-CoV-2 ORF1ab Gene by the Use of Electrochemiluminescent Biosensor Based on Dual-Probe Hybridization

**DOI:** 10.3390/s22062402

**Published:** 2022-03-21

**Authors:** Chunying Jiang, Xihui Mu, Shuai Liu, Zhiwei Liu, Bin Du, Jiang Wang, Jianjie Xu

**Affiliations:** State Key Laboratory of NBC Protection for Civilian, Beijing 102205, China; jiangchunying20@163.com (C.J.); 15155922415@163.com (S.L.); liuzhw07@lzu.edu.cn (Z.L.); dubin51979@163.com (B.D.); roverman@163.com (J.W.); xujianjie@sklnbcpc.cn (J.X.)

**Keywords:** ECL biosensor, dual-probe hybridization, SARS-CoV-2, ORF1ab gene

## Abstract

To satisfy the need to develop highly sensitive methods for detecting the severe acute respiratory syndrome coronavirus type 2 (SARS-CoV-2) and further enhance detection efficiency and capability, a new method was created for detecting SARS-CoV-2 of the open reading frames 1ab (ORF1ab) target gene by a electrochemiluminescence (ECL) biosensor based on dual-probe hybridization through the use of a detection model of “magnetic capture probes—targeted nucleic acids—Ru(bpy)_3_^2+^ labeled signal probes”. The detection model used magnetic particles coupled with a biotin-labeled complementary nucleic acid sequence of the SARS-CoV-2 ORF1ab target gene as the magnetic capture probes and Ru(bpy)_3_^2+^ labeled amino modified another complementary nucleic acid sequence as the signal probes, which combined the advantages of the highly specific dual-probe hybridization and highly sensitive ECL biosensor technology. In the range of 0.1 fM~10 µM, the method made possible rapid and sensitive detection of the ORF1ab gene of the SARS-CoV-2 within 30 min, and the limit of detection (LOD) was 0.1 fM. The method can also meet the analytical requirements for simulated samples such as saliva and urine with the definite advantages of a simple operation without nucleic acid amplification, high sensitivity, reasonable reproducibility, and anti-interference solid abilities, expounding a new way for efficient and sensitive detection of SARS-CoV-2.

## 1. Introduction

The new coronavirus disease 2019 (COVID-19) caused by SARS-CoV-2 has become a global public health threat with its rapid transmission and high mortality rate, causing great concern worldwide. It belongs to the same family as the Severe Acute Respiratory Syndrome (SARS) virus that surfaced in 2003, and the Middle East Respiratory Syndrome (MERS) virus that emerged in 2012 in the Middle East, posing a significant threat to human health [1,2,3,4]. Since early diagnosis and cutting off the transmission route are paramount to preventing and controlling the epidemic, the rapid and accurate detection of SARS-CoV-2 has become a key area of focus [5,6,7].

Nucleic acid detection is currently the gold standard for detecting SARS-CoV-2, and the primary detection tool with the advantages of early diagnosis, high sensitivity, and specificity [8,9]. Current nucleic acid-based assays include complete genome sequence [10,11], real-time polymerase chain reaction (RT-PCR) [12,13], isothermal nucleic acid amplification [14,15], CRISPR [16,17], enzyme-linked immunosorbent assay [18], infrared absorption spectroscopy [19], and biosensor detection [20,21,22]. As the probability of genetic mutation and complexity of SARS-CoV-2 strains increase during the spreading process [23] despite rapid detection through existing methods, there is increased threat to human life and health. It is imperative to obtain high-affinity specific identification probes for conserved sequences, which enable efficient capture and specific identification of target genes, and develop multiple highly sensitive SARS-CoV-2 detection methods and rapid screening tools to further enhance detection efficiency and capability.

**Figure 1:**
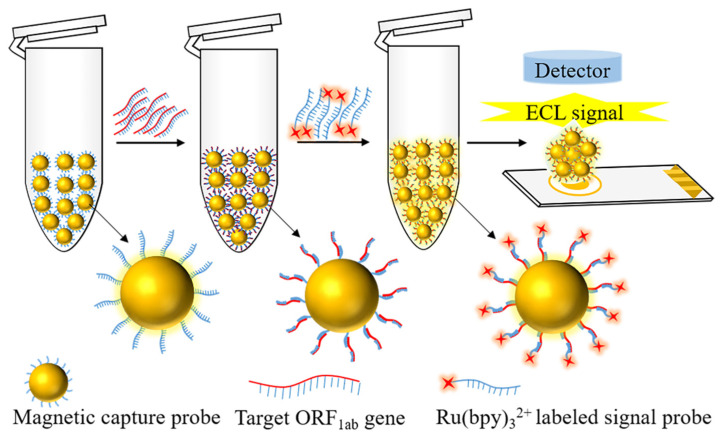
The schematic for detecting SARS-CoV-2 ORF1ab gene through the use of the ECL biosensor, based on dual-probe hybridization.

The ECL biosensor shows broad application prospects in nucleic acid detection of SARS-CoV-2 with advantages of simple operation, high sensitivity, and specificity. There are not many studies on detecting SARS-CoV-2 nucleic acids through the use of ECL biosensor based on screen-printed gold electrodes. How to design specific dual probes for conserved sequences needs to be investigated deeply to improve detection sensitivity. Based on the need to develop a highly sensitive ECL biosensor method for detecting SARS-CoV-2, this study has combined the high specificity of dual-probe hybridization with the advantages of the efficient sensitivity ECL biosensor technology. In our study, specific probes for particular sequences of ORF1ab genes are designed and prepared, and the detection model of magnetic capture probes—targeted nucleic acids—Ru(bpy)_3_^2+^ labeled signal probes is used to establish a new method using an ECL biosensor based on dual-probe hybridization to detect SARS-CoV-2 as shown in Figure 1, which provides a reference for the effective diagnosis of early SARS-CoV-2 infection, timely treatment, and control of disease spread.

## 2. Materials and Methods

### 2.1. Reagents and Instruments

#### 2.1.1. Main Reagents

Binding buffer (pH = 7.4 10 mM Tris-HCl, 1 mM EDTA, 2 M NaCl), PBS (0.01 M, pH = 7.4) and (0.01 M, pH = 8.5) were prepared in our lab. Ru(bpy)_3_^2+^-NHS ester, and bovine serum protein and glycine were purchased from Sigma (St. Louis, MO, USA). The Procell solution composed of Tripropylamine (TPA) was purchased from Beijing Biolot Diagnostics Co., Ltd. (Beijing, China). DMF (*N, N-dimethylformamide)* was purchased from Shanghai Macklin Biochemical Co., Ltd. (Shanghai, China). Streptavidin-modified magnetic particles were purchased from Invitrogen Life Technologies (Oslo, Norway), the diameter of which is 2.8 µm. Influenza A virus subtype H1N1, H5N1, and H7N9 pseudovirus, and SARS, MERS, and SARS-CoV-2 pseudovirus were purchased from Sangon Biotech (Shanghai, China). MiniBEST Viral RNA/DNA Extraction Kit Ver.5.0 was purchased from TaKaRa Bio Inc. (Beijing, China). Saliva and urine were obtained from humans. The target ORF1ab gene and probes were synthesized by Beijing Xingfangyuan Biotechnology Co., Ltd. (Beijing, China).

#### 2.1.2. Main Instruments

UV–vis spectrophotometer was purchased from Thermo Fisher Scientific Inc. (Massachusetts, MA, USA). The HS-3 vertical mixer was purchased from Scientz Biotechnology Co., Ltd. (Ningbo, China), the oscillator was purchased from Eppendorf China Ltd. (Beijing, China), and the magnetic separation frames were purchased from Promega (Beijing) Biotech Co., Ltd. (Beijing, China). The ECL biosensor was designed by our team, and processed by the Xi’an Remex Analysis Instrument Co. Ltd. (Xi’an, China). The screen-printed gold electrode comprised a working electrode, a reference electrode, and a counter electrode. The working electrode and counter electrode material were gold, and the reference electrode was a mixture of silver chloride silver. The electrode size is 50 mm long and 12.6 mm wide, purchased from Zensor Technology Co., Ltd. (Taiwan, China).

### 2.2. Experimental Methods

#### 2.2.1. Design and Synthesis of the Sequences of Biotin-Modified Probes (Biotin Probes) and Amino-Modified Probes (Amino Probes)

The website of BLAST was used to compare the sequences of SARS-CoV-2 genes. According to the results, the conserved region fragment of the ORF1ab gene was selected as the target region. The sequences of biotin probes and amino probes that can be hybridized with target ORF1ab genes were designed by the use of the Primer Express 5.0 software. The target ORF1ab gene and probes were synthesized by Beijing Xingfangyuan Biotechnology Co., Ltd. (Beijing, China), and the sequences are provided in Table 1.

#### 2.2.2. Preparation of Magnetic Capture Probes

The magnetic beads (200 µL, 10 mg/mL) were washed with binding buffer by rotating them for 5 min in a vertical mixer. Magnetic beads were separated, and the supernatant was discarded. The washing process was repeated three times. The volume of magnetic beads was made 1 mL with binding buffer. Then the magnetic beads were mixed with a certain amount of biotin probes, incubated, shaken for 10 min, and washed three times with binding buffer. Next, PBS (0.01 M, pH = 7.4), including 1% BSA and 1% glycine, were added to close the uncombined site of the magnetic beads for 30 min. Finally, the magnetic capture probes were washed and dispersed in 200 µL of binding buffer for the following reaction.

#### 2.2.3. Preparation of Ru(bpy)_3_^2+^ Labeled Signal Probes

Amino probes (176 µL, 100µM) dissolved by PBS (0.01 M, pH = 8.5) were mixed with 20 µL of DMF solution containing 10 mg/mL Ru(bpy)_3_^2+^-NHS ester and reacted in an oscillator with an oscillation rate of 600 rpm/min for 12 h. After that, the above solution was taken out, and the volume made 1 mL with PBS (0.01 M, pH = 7.4) and centrifuged at 8000 *g*/min for 30 min. This was repeated three times to prepare Ru(bpy)_3_^2+^ labeled signal probes for the subsequent reaction in a fixed volume of 140 µL.

#### 2.2.4. Development of a Method for the Detection of SARS-CoV-2 through the Use of an ECL Biosensor Based on Dual-Probe Hybridization

Firstly, 10 µL of different concentrations of the target ORF1ab gene (0.1 fM, 1 fM, 100 fM, 10 pM, 1 nM, 100 nM, 1 µM, 10 µM, 100 µM) were added to 40 µL of magnetic capture probes respectively, incubated for 10 min, washed three times with PBS (0.01 M, pH = 7.4), and the volume made 1 mL with PBS. Then the above substances were mixed with 2.5 µL of Ru(bpy)_3_^2+^ labeled signal probes, incubated for 10 min, washed with PBS. After that, the hybridization complexes of magnetic capture probes—targeted nucleic acids—Ru(bpy)_3_^2+^ labeled signal probes were prepared. Finally, 200 µL of TPA was added to the hybridization complexes. ECL tests use the optical method to detect the variation real time response curve of chemiluminescence and intensities produced by Ru(bpy)_3_^2+^. As for the test, 10 µL of the hybridization complexes were added to the ECL chip each time, and the ECL intensity was measured by cyclic voltammetry scanning at 0.2–1.35 V, and the rate is 0.1 V/S. A standard curve was established with the logarithm of the concentration of the ORF1ab gene (X = LgC, µM) as the abscissa and its ECL intensity as the ordinate (Y, a.u.). The linear range and LOD were determined according to a standard curve for detecting the SARS-CoV-2 ORF1ab target gene through the use of an ECL biosensor based on dual-probe hybridization.

### 2.3. Reproducibility and Specificity Examination

In the linear range of detection, the method of reproducibility through the use of an ECL biosensor based on dual-probe hybridization was examined by detecting different concentrations of ORF1ab target genes (10 pM, 10 nM, 10 µM). Furthermore, the method of specificity was examined by detecting 10 pM concentrations of influenza A virus subtype H1N1, H5N1, and H7N9 pseudovirus, SARS, MERS, and SARS-CoV-2 pseudovirus.

### 2.4. Detection of Simulation Samples

SARS-CoV-2 pseudovirus (180 µL) was added to saliva and urine (20 µL) to prepare simulation samples. After nucleic acid extraction, the concentration of extracts (10 fM) was obtained. Then the above dual-probe hybridization reaction was performed, and the ECL intensity was measured. The recovery rate and relative standard deviation (RSD) were calculated according to the standard curve.

## 3. Results and Discussion

### 3.1. Preparation of Magnetic Capture Probes and Determination of the Optimal Immobilization Amount of Biotin Probes

**Figure 2:**
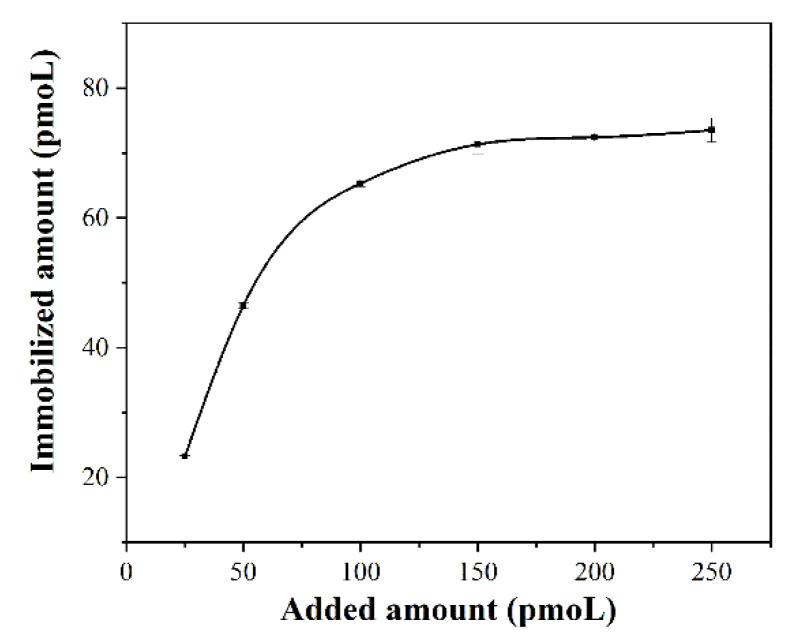
Determination of the optimal immobilized amount of biotin probes on the surface of the magnetic particles.

Since the streptavidin-modified magnetic particles can be specifically bound with biotin probes, the magnetic capture probes were prepared by immobilizing the biotin probes onto the surface of the magnetic particles. The magnetic capture probes were prepared by adding different concentrations of the biotin-probe solution to 200 µg of streptavidin-modified magnetic particles. The optional amount of biotin probes immobilized on 200 µg magnetic particles was calculated by measuring the absorbance value of the biotin-probe solution at A260 nm before and after binding, according to the formula binding rate = (A260 nm pre − A260 nm post)/(A260 nm pre) × 100%, respectively. The results from Table 2 and Figure 2 show that the amount of biotin probes immobilized on the surface of magnetic particles gradually increased, and saturated with the increase in amount of biotin probes added. It was confirmed that the optimal added amount of biotin probes per 200 µg magnetic particles was determined as 150 pmoL, and the amount of immobilized biotin probes per 200 µg magnetic particles was 71.32 pmoL.

### 3.2. Preparation and Characterization of Ru(bpy)_3_^2+^ Labeled Signal Probes

**Figure 3:**
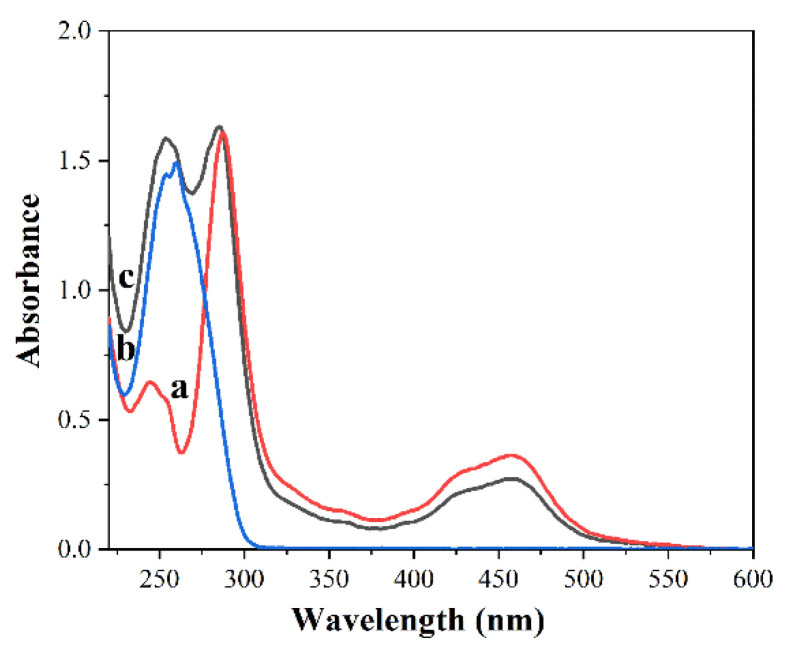
The UV–vis spectrum of the solution Ru(bpy)_3_^2+^-NHS ester labeled the amino probes. Curve a is the spectrum of Ru(bpy)_3_^2+^-NHS ester. Curve b is the spectrum of amino probes. Curve c is the spectrum of Ru(bpy)32+ labeled signal probes.

Ru(bpy)_3_^2+^ labeled signal probes are prepared by reacting Ru(bpy)_3_^2+^-NHS ester with amino probes and analyzed UV–vis spectrum. Figure 3 shows the UV–vis spectrum of the solution before and after Ru(bpy)_3_^2+^-NHS ester labeled the amino probes. Curve a is the spectrum of Ru(bpy)_3_^2+^-NHS ester, and the peak at the wavelength of 247 nm, 287 nm, and 458 nm are the three characteristic absorption peaks of Ru(bpy)_3_^2+^-NHS ester. Curve b is the spectrum of amino probes, and the peak at the wavelength of 260 nm is the characteristic absorption peak of amino probes. Curve c is the spectrum of Ru(bpy)_3_^2+^ labeled signal probes, and the peak at the wavelength of 260 nm, 287 nm, and 458 nm are the characteristic absorption peaks of Ru(bpy)_3_^2+^ labeled signal probes, thus indicating that Ru(bpy)_3_^2+^ has been successfully labeled on amino probes.

### 3.3. Linearity Range and LOD

**Figure 4:**
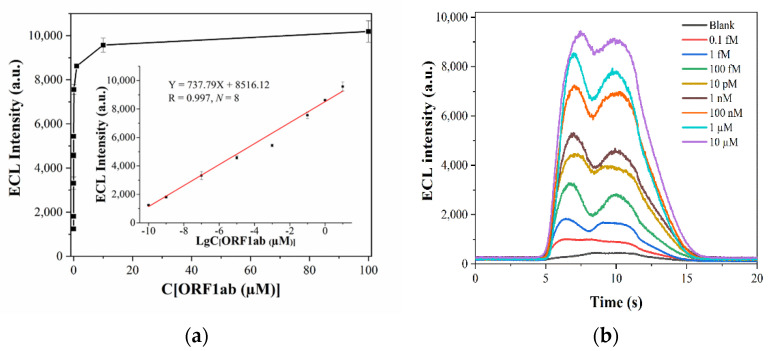
(**a**) Standard curve of detecting different concentrations of the nucleic acid of SARS-CoV-2 using an ECL biosensor based on dual-probe hybridization. (**b**) The response curve of detecting different concentrations of the nucleic acid of SARS-CoV-2 using an ECL biosensor based on dual-probe hybridization.

Hybridization complexes bound with different concentrations of ORF1ab target gene (0.1 fM, 1 fM, 100 fM, 10 pM, 1 nM, 100 nM, 1 µM, 10 µM, 100 µM) were detected by using an ECL biosensor. The ECL intensity was obtained, the detection process repeated five times, and the average value of the ECL intensity taken for data analysis. When the ORF1ab target gene concentrations were in the range of 0.1 fM–10 µM, a significant linear relationship was found between the logarithm of the concentration of the ORF1ab target gene (X = LgC, µM) as the abscissa and its ECL intensity as the ordinate (Y, a.u.). The regression equation was Y = 737.79X + 8516.12 (R = 0.997, *N* = 8). The measured ECL intensity of 0.1 fM was 1241.2 ± 48.35, and the ECL intensity of the blank was 405.2 ± 11.77. The LOD was determined as 0.1 fM by taking S/N ≥ 3 as the LOD determination standard, as shown in Figure 4a,b.

### 3.4. Reproducibility and Specificity Examination

**Figure 5:**
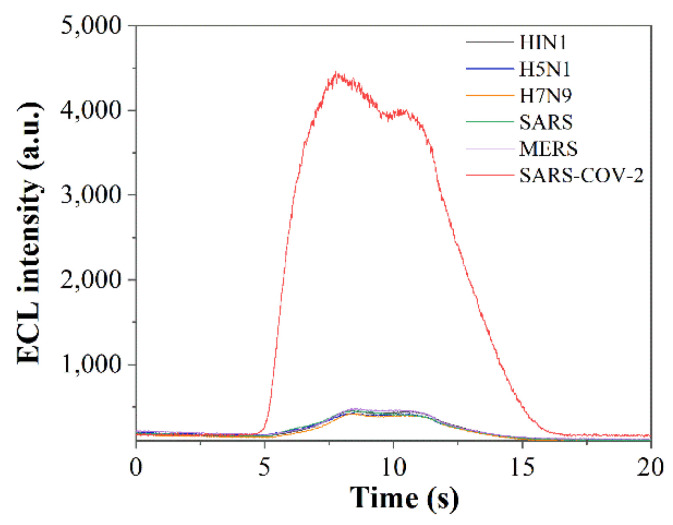
Response curves of detecting different viruses using an ECL biosensor based on dual-probe hybridization.

In the linear range of the detection, the concentrations of the ORF1ab target gene at concentrations of 10 pM, 10 nM, and 10 µM were selected for the above dual-probe hybridization reactions. The ECL intensity was measured five times for each one, and the averaged ECL intensity was obtained as 4523.4 ± 87.32, 6432.6 ± 273.21, and 9710.6 ± 151.85, respectively. The RSDs were 1.93%, 4.25%, and 1.56%, respectively, which indicated that the method has reasonable reproducibility. Influenza A virus subtype H1N1, H5N1, H7N9 pseudovirus, SARS, MERS, and SARS-CoV-2 pseudovirus were selected for specificity assay. The concentration of extracts (10 pM) was obtained after extraction of nucleic acids, and the above dual-probe hybridization reaction was performed. ECL detection was performed five times, and the ECL signals were 416.6 ± 38.02, 441.4 ± 16.95, 432.6 ± 31.64, 443 ± 19.42, and 441.7 ± 26.10, 4569.4 ± 119.85 with RSDs of 9.13%, 3.84%, 7.31%, 4.38%, and 5.91%, respectively, and response signals as shown in Figure 5. This method can overcome the interference of frequent influenza viruses in the autumn and winter seasons to detect SARS-CoV-2, and can accurately distinguish influenza, SARS, and MERS viruses from SARS-CoV-2, indic0000ating that it has reasonable specificity.

### 3.5. Simulated Sample Determination

Upon testing the simulated samples, 10 fM of SARS-CoV-2 pseudovirus in saliva and urine was detected, as shown in Table 3. After five times of measurements and data calculations, the recovery ratios were 94.83% and 93.65%, and RSDs were 3.22% and 3.67%, respectively. It can meet the requirements of testing simulated samples in saliva and urine.

## 4. Conclusions

This study combined the advantages of dual-probe hybridization’s high specificity with a highly sensitive ECL biosensor. This research has established a new method of using the ECL biosensor, based on dual-probe hybridization, to detect SARS-CoV-2, achieving highly sensitive detection of the ORF1ab gene of SARS-CoV-2, with a linear range of 0.1 fM-10 µM, regression equation of Y = 737.79X + 8516.12 (R = 0.997, *N* = 8), and LOD of 0.1 fM. The method can meet the requirements for analyzing simulated samples such as saliva and urine, with the advantages of high sensitivity, stable reproducibility, and anti-interference solid abilities. Compared with the existing reported detection method for the SARS-CoV-2 gene by ECL biosensor based on the amplification technique [24,25] and other biosensors [26,27,28,29,30,31], the ECL biosensor based on dual-probe hybridization detection of SARS-CoV-2 has improved sensitivity, as shown in Table 4. It also has the advantages of simplifying the operation steps without amplifying and shortening the detection time, achieving rapid and highly sensitive detection within 30 min. This method provides a promising new way for the highly sensitive detection of SARS-CoV-2.

## Figures and Tables

**Table 1:** Target gene and probes’ sequences.

Name	Sequences (5′-3′)
Target ORF1ab gene	CTCACCTTATGGGTTGGGATTATCCTAAATGTGATAGAGCCATGCCTAACATGCTTAGAATTATGGCCTCACTTGTTCTTGCTCGCAAACATACAACGTGTTGTAGCTTGTCACACCGTT
Biotin probes	GCATGGCTCTATCACATTTAGGA-bio
Amino probes	NH_2_- TGCGAGCAAGAACAAGTGAGG

**Table 2:** The absorbance value of biotin-probe solution before and after binding to the magnetic particle.

Added Amount (pmoL)	A_260 nm_ Pre	A_260 nm_ Post	Binding Rate (%)	Immobilized Amount (pmoL)
25	0.0797 ± 0.0020	0.0057 ± 0.0006	92.89	23.22 ± 0.15
50	0.1507 ± 0.0015	0.0107 ± 0.0012	92.92	46.46 ± 0.36
100	0.3387 ± 0.0006	0.1175 ± 0.0021	65.31	65.31 ± 1.55
150	0.5700 ± 0.0056	0.2990 ± 0.0046	47.54	71.32 ± 1.55
200	0.7530 ± 0.0017	0.4803 ± 0.0012	36.21	72.42 ± 0.60
250	0.8537 ± 0.0032	0.6027 ± 0.0045	29.97	73.50 ± 1.87

**Table 3:** Simulated sample determination results.

Sample Type	Added Amount (fM)	Detectable Amount (fM)	Recovery Ratio (%)	RSD(%)
Saliva	10	9.48 ± 0.30	94.83%	3.22%
Urine	10	9.36 ± 0.34	93.65%	3.67%

**Table 4:** Different types of biosensors detect SARS-CoV-2.

Biosensor Type	Detection Method	Target	LOD	References
Fluorescent Bioplatform	Magnetic nanomicrospheres	RNA	16.61 fM	[26]
Surface plasmon resonance biosensor	Gold Nano Island	N gene	0.125 fM	[27]
		E gene	0.451 fM	
Field-effect transistor nanosensor	Morpholino-modified graphene	RNA	0.37 fM	[28]
	carbon nanotube	RdRp gene	10 fM	[29]
Electrochemical biosensor	TdT-mediated DNA polymerization	RNA	26 fM	[30]
	Polyaniline nanowires	N gene	3.5 fM	[31]
ECL biosensor	DNA walker amplification	RdRp gene	0.21 fM	[24]
	Entropy-driven amplification	RdRp gene	2.67 fM	[25]
	AuNMs and CDs	ORF1ab gene	0.514 fM	[32]
	Dual-probes hybridization	ORF1ab gene	0.1 fM	This work

## Data Availability

Not applicable.
